# Supplementary material for: Using Multiplexed CRISPR/Cas9 for Suppression of Cotton Leaf Curl Virus
Source: Int J Mol Sci. 2021 Nov 21;22(22):12543. doi: 10.3390/ijms222212543 (PMC8618328; doi:10.3390/ijms222212543)
Supplement: Supplementary file 1 [file ijms-22-12543-s001.zip › ijms-1423361-supplementary.pdf]

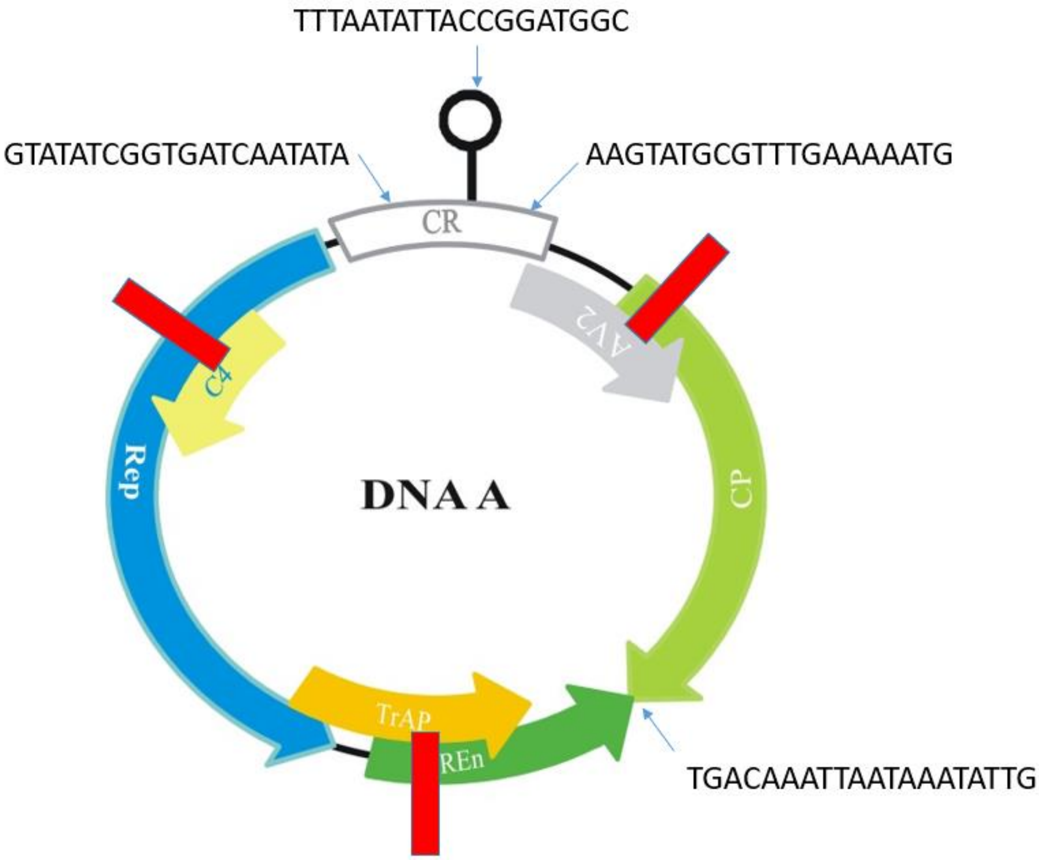

Figure S1. Map of DNA-A of CLCuV. The map is showing different target sites in the DNA-A of the virus which were screened to find out potential target sites for viral interference. Red bars showing consensus sequences selected in the coding regions to target six viral genes with three gRNAs simultaneously.

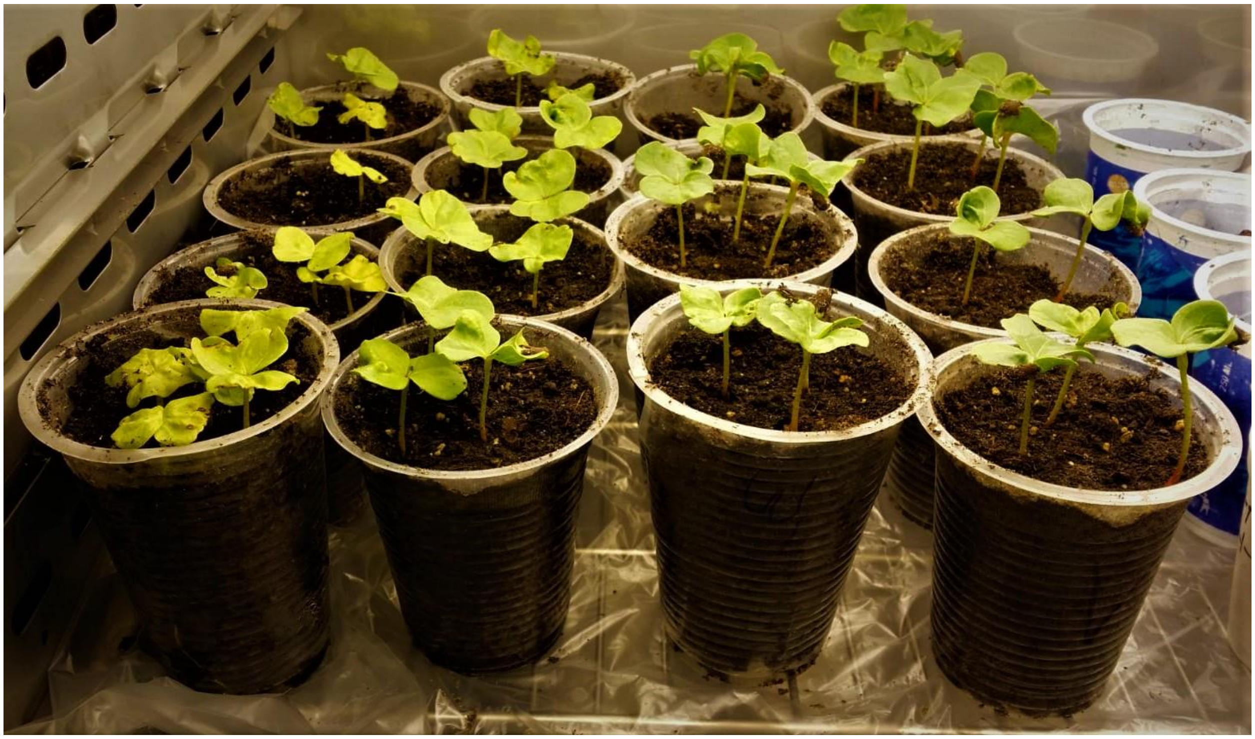

Figure S2. Cotton seedlings after 10 days of sowing, ready for transformation. The seedlings were grown at 25 ^{0}C under dark in plastic pots having peat moss.

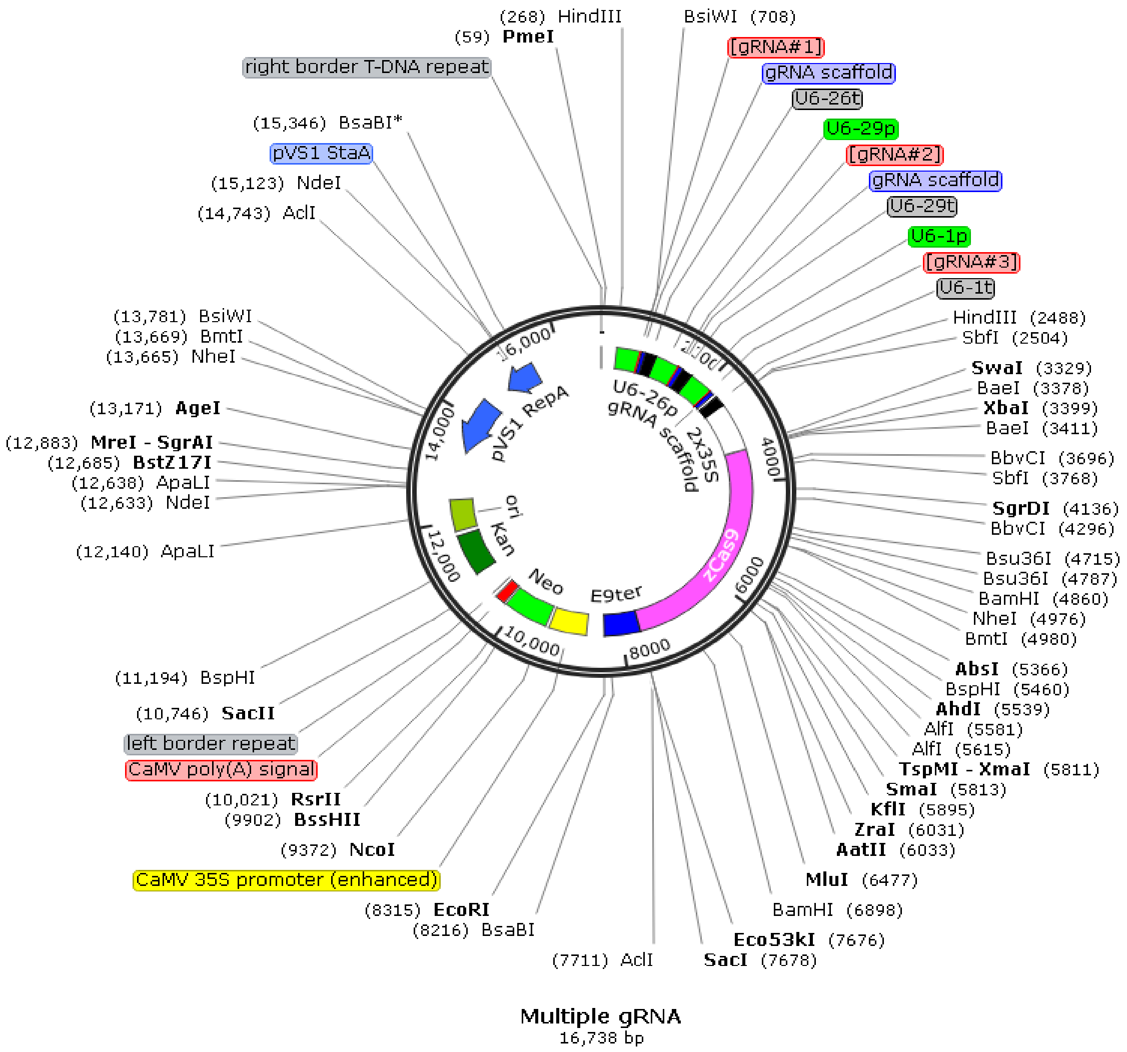

Figure S3. Map of Multiple gRNA Cas9 expression vector. zCas9 was expressed in plants under 2x35S promoter. The w3 gRNAs under were cloned under three different promoters (derived from Arabidopsis) to target multiple sites in the CLCuV to develop broad-spectrum resistance against CLCuD. Neomycin was used as plant selection marker while kanamycin was used as bacterial selection marker.

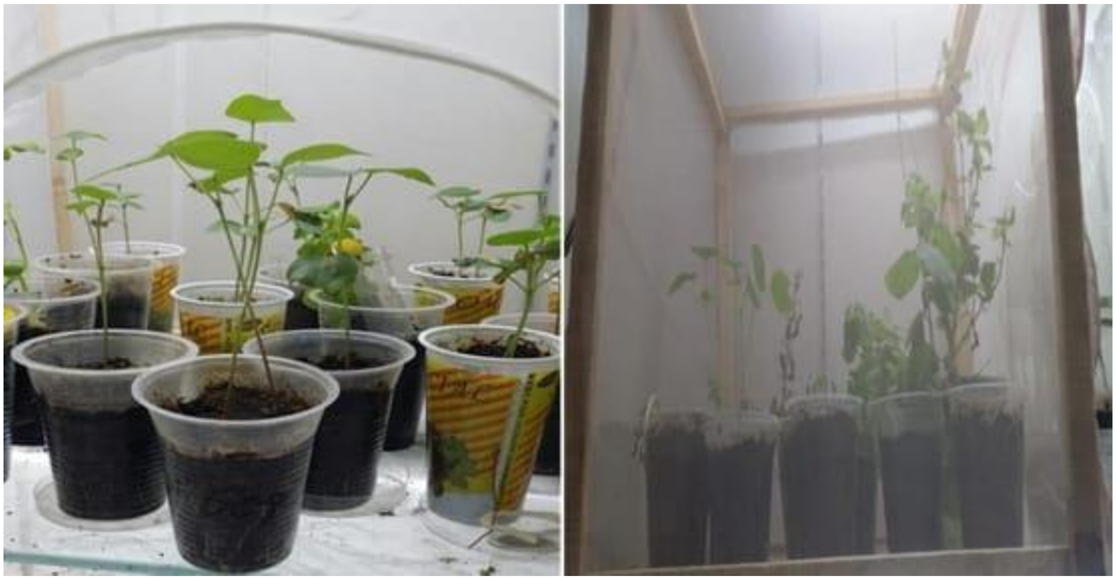

Figure S4. Challenging plants with infected whitefly. Cotton seedlings were challenged with infected whiteflies in the net cages. The area of each cage was 4 square feet. Initially 30-50 whiteflies were left in each cage for infecting the plants. The data from 14 to 21 days post infection was taken to evaluate the multiplex CRISPR for virus resistance

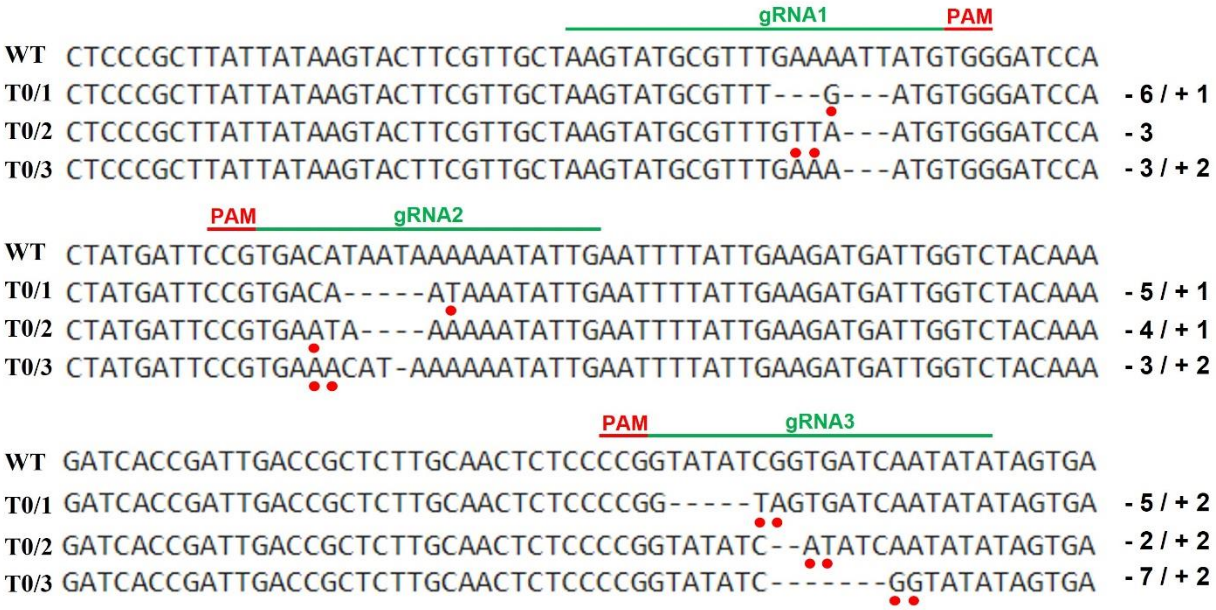

Figure S5: CRISPR-induced mutation analysis of CLCuV DNA-A in virus infected plants. Indels in viral genome were observed after sequence alignment of wild-type and multiplexed CRISPR-edited DNA-A encompassing gRNA target sequence. Dashes indicating deletion of bases compared with wild type sequence and red dots indicating addition of new bases. Red underline representing PAM region and green underline representing 20 bp gRNA sequence.

Table S1: Evaluation of multiplex CRISPR/Cas for suppression of CLCuV in *N. benthamiana*

Inoculum	Experiment No.	No. of plants	Symptomatic plants	% symptomatic plants	Symptoms severity	Delay in symptoms (No. of days)	Plants recovered at 30 dpi	Diagnostics	
								PCR	qPCR
Non-inoculated plants	In all experiments	3	N/A	N/A	N/A	N/A	N/A	N/A	N/A
CLCuV/CLCuMB	1	10	10	100	Severe	-	No	+10/10	1
	2	10	10	100	Severe	-	No	+10/10	1
	3	10	10	100	Severe	-	No	+10/10	1
Cas9-gRNA+ CLCuV/CLCuMB	1	10	4/10	40	Mild	2-5	Yes	+8/10	0.32
	2	10	3/10	30	Mild	2-5	Yes	+6/10	0.36
	3	10	5/10	50	Mild	2-5	Yes	+9/10	0.41

Table S2: Evaluation of multiplex CRISPR/Cas for suppression of CLCuV in cotton

Inoculum	Experiment No.	No. of plants	Symptomatic plants	% symptomatic plants	Symptoms severity	Delay in symptoms (No. of days)	Plants recovered at 30 dpi	Diagnostics	
								PCR	qPCR
Non-inoculated plants	In all experiments	3	N/A	N/A	N/A	N/A	N/A	N/A	N/A
CLCuV/CLCuMB	1	10	10	100	Severe	-	No	+10/10	1
	2	10	10	100	Severe	-	No	+10/10	1
	3	10	10	100	Severe	-	No	+10/10	1
Cas9-gRNA+ CLCuV/CLCuMB	1	10	4/10	40	Mild	3-4	Yes	+8/10	0.38
	2	10	5/10	50	Mild	2-4	Yes	+6/10	0.40
	3	10	3/10	30	Mild	3-5	Yes	+9/10	0.31

Table S3: Transgenic plants developed from hypocotyls (Coker 312)

Experiment No.	No. of explants used (hypocotyls)	Gene Construct	Plants developed
1	480	CRISPR/Cas9	1
2	525	CRISPR/Cas9	2
3	460	CRISPR/Cas9	2
